# An overview of tissue engineering approaches for management of spinal cord injuries

**DOI:** 10.1186/1743-0003-4-15

**Published:** 2007-05-14

**Authors:** Ali Samadikuchaksaraei

**Affiliations:** 1 Consultant in Tissue Engineering and Regenerative Medicine, Consultant in General Medicine, Assistant Professor, Department of Biotechnology, Faculty of Allied Medicine and Cellular and Molecular Research Center, Iran University of Medical Sciences, Iran

## Abstract

Severe spinal cord injury (SCI) leads to devastating neurological deficits and disabilities, which necessitates spending a great deal of health budget for psychological and healthcare problems of these patients and their relatives. This justifies the cost of research into the new modalities for treatment of spinal cord injuries, even in developing countries. Apart from surgical management and nerve grafting, several other approaches have been adopted for management of this condition including pharmacologic and gene therapy, cell therapy, and use of different cell-free or cell-seeded bioscaffolds. In current paper, the recent developments for therapeutic delivery of stem and non-stem cells to the site of injury, and application of cell-free and cell-seeded natural and synthetic scaffolds have been reviewed.

## Introduction

Spinal cord injury (SCI) usually leads to devastating neurological deficits and disabilities. The data published by the National Spinal Cord Injury Statistical Center in 2005 [1] showed that the annual incidence of SCI in the United States is estimated to be 40/milliion. It also estimated that the number of patients with SCI in US was estimated to be 225,000 to 288,000 persons in July 2005 (see Ackery *et al *[2] for a review on the worldwide epidemiology of SCI).

It has been shown that patients with SCI have more depressive feelings than general population [3]. The marriage of patients who are married at the time of injury is more likely to be compromised than general population. Also, the likelihood of getting married after the injury is lower than the general population [1]. In addition, there are significant reductions in rates of occupation and employment after injury, especially during the first year [4].

In addition, tremendous costs are imposed on community by the spinal cord injury. The costs include cost of initial and subsequent hospitalizations, rehabilitation and supportive equipment, home modifications, personal assistance, institutional care and loss of income. It has been shown that the average initial hospital expenses for a patient with SCI is around $95000 and the average yearly expenses after recovery and rehabilitation is around $14135 [5]. The average lifetime cost that is directly attributed to SCI is estimated to be $620000–$2800000 for each patient aged 25 years at the time of injury, and $450000–1600000 for each patient aged 50 at the time of injury [1].

These data show that apart from the patients, SCI imposes high psychosocial and financial costs to the family of the patient and to the community. Therefore, investment for the development of any treatment modality that improves patients' signs and symptoms, and subsequently, diminishes the health care costs of SCI is quite justifiable.

## Pathophysiology

The neurological damage that is incurred at the time of mechanical trauma to the spinal cord is called "primary injury". The primary injury provokes a cascade of cellular and biochemical reactions that leads to further damage. This provoked cascade of reactions is called "secondary injury".

Primary injury occurs following (1) blunt impact, (2) compression, and (3) penetrating trauma. Blunt impacts can lead to concussion, contusion, laceration, transection or intraparenchymal hemorrhage. Cord compression usually results from hyperflexion, hyperextension, axial loading, and severe rotation [6]. Gunshot and stab wounds are examples of penetrating traumas. The immediate mechanical damage to the neurons leads to the cell necrosis at the point of impact [7].

Several mechanisms are involved in secondary injury of which, vascular changes at the site of injury are the most important events. The microvascular alterations include loss of autoregulation, vasospasm, thrombosis, hemorrhage and increased permeability. These, in combination with edema, lead to hypoperfusion, ischemia and necrosis [8]. Other major mechanisms include: (1) free radicals formation and lipid peroxidation [9] (2) accumulation of excitatory neurotransmitters, e.g. glutamate (acting on N-methyl-D-aspartate [NMDA] and non-NMDA receptors), and neural damage due to excessive excitation (excitotoxicity) [10] (3) loss of intracellular balance of sodium, potassium, calcium and magnesium and subsequent increased intracellular calcium level [11] (4) increased level of opioids, especially dynorphins, at the site of injury, which contribute to the pathophysiology of secondary injury [12,13] (5) depletion of energy metabolites leading to anaerobic metabolism at the site of injury and increasing of LDH activity [14] (6) provocation of an inflammatory response and recruitment and activation of inflammatory cells associated with secretion of cytokines, which contribute to further tissue damage [15], and (7) activation of calpains [16] and caspases and apoptosis [17,18].

Primary and secondary injuries lead to the cell loss in the spinal cord. In penetrating injuries, this leads to scarring and tethering of the cord [7]. Demyelination occurs following the loss of oligodendrocytes, which causes conduction deficits [19]. In contusion injuries, a cystic cavity surrounded by an astrocytic scar is formed following this tissue loss. Where the injury extends to pia mater, collagen will also contribute in the formation of the scar tissue. As a physical barrier, the scar dos not allow the axons to grow across the cavity [20].

Crushed or transected nerve fibers exhibit regenerative activities by outgrowth of neurites. This is called regenerative sprouting. But, this would not be more than 1 mm, because there are inhibitory proteins in the CNS that inhibit this activity [21]. Among these inhibitory proteins, the myelin proteins Nogo and MAG could be named, which are exposed after the injury [22,23]. Inhibitory proteins have been identified in the extracellular matrix of the scar tissue as well, mainly chondroitin sulfate proteoglycans (CSPGs) secreted by reactive astrocytes [7,24]. Permanent hyperexcitability is another mechanism that develops in many cells leading to different signs and symptoms [7].

## Approaches to treatment

Stabilization of the spine and restoration of its normal alignment together with surgical decompression of the cord is the subject of individual or institutional preferences; and there is no consensus regarding necessity, timing, nature, or approach of surgical intervention [25,26].

There have been several attempts to target and modulate the mechanisms leading to the secondary injury by pharmacological interventions (see Sayer *et al *[27] and Baptiste and Fehlings [28] for review), neutralization of the effects of regenerative sprouting inhibitory proteins (see Scott *et al *[29] for review) and gene therapy (see Blits and Bunge [30] and Pearse and Bunge [31] for review).

The core approach of tissue engineering consists of provision of an interactive environment between cells, scaffolds and bioactive molecules to promote tissue repair. To achieve this goal, the *ex vivo *engineered cell-scaffold constructs could be transplanted to the site of injury. Alternatively, the repair is achieved by delivery of scaffold-free cells or acellular scaffolds to the damaged tissue.

## Cell therapy

### Macrophages

Due to the immune privilege, recruitment of macrophages is limited in CNS and the resident microglia cells are the main immune cells that are activated after SCI [19]. It has been shown that controlled boosting of local immune response by delivering of autologous macrophages, which were alternatively activated to a wound-healing phenotype, can promote recovery from the spinal cord injury. Initial experiments with implantation of macrophages activated by preincubation with peripheral nerve fragments lead to partial recovery of paraplegic rats [32]. Improved motor recovery and reduced spinal cyst formation of rats was also observed by implantation of macrophages activated by incubation with autologous skin [33]. The postulated mechanisms are activation of infiltrating T cells, and increased production of trophic factors such as brain-derived neurotrophic factor (BDNF) [33,34] leading to removal of inhibitory myelin debris [32]. Promotion of a permissive extracellular matrix containing laminin is another observation [34]. Following these and subsequent positive results from animal experiments, autologous macrophages activated by incubation with autologous skin, under the brand name of ProCord, were entered into a multicentric clinical trial. The results of phase I studies show that out of eight patients in the study, three recovered clinically significant neurological motor and sensory function. Also, it has been shown that this cell therapy is well tolerated in patients with acute SCI [35].

### Dendritic cells

In animal model studies, transplantation of dendritic cells into the injured spinal cord of mice led to better functional recovery as compared to controls [36]. The implanted dendritic cells induced proliferation of endogenous neural stem/progenitor cells (NSPCs) and led to de novo neurogenesis. This observation was attributed to the action of secreted neurotrophic factors such as neurotrophin-3, cell-attached plasma membrane molecules, and possible activation of microglia/macrophages by implanted dendritic cells [36].

Dendritic cells pulsed (incubated) with encephalitogenic or non-encephalitogenic peptides derived from myelin basic protein when administered intravenously or locally to the site of injury, promoted recovery from SCI [37]. The mechanisms proposed to explain this phenomenon is based on presentation of the loaded antigen to the naïve T cells by dendritic cells. The stimulated T cells start a cascade of events leading to "beneficial autoimmunity". They may secrete growth factors that protect the injured tissue. Also, they lead to a transient reduction in the nerve's electrophysiological activities, decreasing nerve's metabolic requirements and thus preserving neuronal viability [38]. This explanation is in line with the finding that in those rats, which are unresponsive to myelin self-antigens, the outcome of CNS injury is worse than normal rats [39].

### Olfactory ensheathing cells (OECs)

Olfactory ensheathing cells (OECs) are glial cells ensheathing the axons of the olfactory receptor neurons. These cells have properties of both Schwann cells and astrocytes, with a phenotype closer to the Schwann cells [40]. OECs can be obtained from olfactory bulb or nasal mucosa (lamina propria). Cells from both sources have been used for treatment of spinal cord injury in animal models. Those from olfactory bulb origin lead to axonal regeneration and functional recovery after transplantation to animals with transected [41,42], hemisected [43,44] or contused [45] spinal cords. Similar results were also obtained by transplantation of OECs isolated from lamina propris in both transected [46] and hemisected [47] models. It has been shown that these cells are able to retain their regenerative ability after cryopreservation [48] and after establishment of a clonally derived cell line [49]. Boosting of regenerative capability of OECs by overexpression of brain-derived neurotrophic factor (BDNF) [50] or glial cell line-derived neurotrophic factor (GDNF) [51] was also tried successfully in animal models.

OECs migrate after implantation [52], decrease neuronal apoptosis [53] and secrete a number of extracellular matrix molecules such as type IV collagen, and the chondroitin sulfate proteoglycan NG2 [54]. They also secrete trophic factors such as vascular endothelial growth factor (VEGF) [54], nerve growth factor (NGF), and BDNF [55]. Remyelination is also increased after transplantation of OECs [56-58]. A comparison of acute versus delayed transplantation of OECs has shown that acute transplantation leads to earlier recovery and better functional and histological results [59]. The efficacy and behavior of olfactory bulb-derived cells were compared with lamina propria (LP)-derived cells after implantation. LP-derived cells showed superior ability to migrate within the spinal cord, and reduce the cavity formation and lesion size, but they enhanced autotomy [60]. All the above properties can explain the observed histological and functional improvements following transplantation of olfactory ensheathing cells to the site of injury.

According to the promising results obtained from animal experiments, several clinical trials have been started. In a large series more than 400 patients underwent transplantation of fetal olfactory bulb-derived cells, of which the results of 171 operations were published [61], showing functional recovery, regardless of age and as early as the first day after implantation [61]. But, an independent observational study of 7 cases from this series did not report any clinically useful sensorimotor, disability, or autonomic improvements [62]. In a recent case report, a rapid functional recovery was noted within 48 hours of transplantation of olfactory bulb-derived cells [63]. This reemphasizes the need for further studies into the mechanism of action of these cells, as according to the animal studies, such a rapid start of improvement is not expected. Nasal mucosal-derived OECs were also used in a phase I clinical trial conducted on 3 patients who were followed for one year after transplantation [64]. The results confirm the safety and feasibility of this approach.

### Schwann cells (SCs)

Schwann cells originating from dorsal and ventral roots are one of the cellular components that migrate to the site of tissue damage after spinal cord injury [65-68]. The remyelinating capability of Schwann cells has been demonstrated in a number of studies [66,69] and the functioning status of this myelin in conduction of neural impulses was confirmed [70,71]. SCs promote axonal regeneration by secretion of adhesion molecules such as L1 and N-CAM, extracellular matrix molecules such as collagen [72] and laminin (see Chernousov and Carey [73] for review), and a number of trophic factors such as FGF-2 [74], nerve growth factor (NGF), brain-derived neurotrophic factor (BDNF) and NT3 (see Mirsky *et al *[75] for review). In addition to their on neural regeneration and remyelination, a number of unwanted effects were also reported following the use of these cells. It has been shown that when SCs come into contact with CNS astrocytes, their migration into the CNS is stopped [76]. Also, corticospinal tracts (CST) show a delayed and poor regenerative activity in response to Schwann cells implantation when compared with OECs [77]. The other unwanted issue in regard to SCs is that the damaged axons, which are stimulated by these cells to regenerate, grow into the grafted population of Schwann cells, but there is little evidence to support that they leave these cells and re-enter their original white matter pathways [70]. When combining SCs transplantation with delivering of neurotrophic factors [78] or OECs plus chondroitinase [79] exit of regenerating axons could be observed from the transplanted population of grafted cells.

In animal model studies, Schwann cells are isolated from either newborn or adult sciatic nerve and cultured in the presence of mitogens. Upon transplantation to the damaged spinal cord of adult animals, they stimulate tissue repair by causing regenerating axons and astroglia to express developmentally related molecules. When compared with the effects of OECs in an acute SCI setting, it was concluded that the degree of functional recovery achieved by SCs is less than OECs [80]. It has been shown that delayed transplantation leads to a higher survival of SCs in host tissue as compared with acute transplantation; meanwhile, implanted Schwann cells cause extensive infiltration of endogenous SCs to the site of injury [81]. Schwann cells are usually transplanted by direct injection to the site of injury, which can add to the inflammatory process in the region. Recently, as an alternative route, transplantation to the subarachnoid space was tried and led to a favorable outcome [82]. The results of a phase I human clinical trial in patients with chronic SCI will be presented in the next annual meeting of the Congress of Neurological Surgeons in Chicago [83].

### Neural stem cells in CNS

Neural stem cells (NSCs) are present in adult and developing central nervous system of mammals and can be isolated and expanded *in vitro *[84]. Neurosphere technique is the most common method for isolation of NSCs. Using this technique, stem cells have been isolated from developing spinal cord [85], cerebral cortex [86] and brain [87], and from adult subependymal, subventricular zone of the lateral ventricle [88,89], cerebral cortex [90] and spinal cord [91]. Also, there was a widely held assumption that dentate gyrus of the hippocampus contains neural stem cells in adults. But, it has been shown recently that dentate gyrus is a source of neural-restricted progenitors (NRPs) and not multipotent stem cells [92]. NRPs are different from neural stem cells as they are committed to neural lineage at time of isolation. It has been shown that NSCs differentiate to neural and glial cells both *in vitro *[93,94] and *in vivo *[93-95]. Also, following a clonal study, it has been reported that neural stem cells from the adult mouse brain can contribute to the formation of chimeric embryos and give rise to cells of all germ layers [96].

The fate of *in vivo *differentiation of neural stem cells depends on the niche they have been transplanted to. When transplanted into a neurogenic region e.g. dentate gyrus [95,97] or subventricular zone [97], they will differentiate into neurons. Transplantation into other, so called, non-neurogenic regions, such as spinal cord [94], will induce them to differentiate into glial cells. Although a few studies report limited differentiation in non-neurogenic regions [84,85], most reports are consistent with differentiation into glial fate. This shows the importance of environmental cues in directing the differentiation of NSCs. NRPs isolated from fetal spinal cord were transplanted into normal and injured spinal cord and differentiated into neurons in normal cords. But, the injured spinal cord niche restricted their differentiation and the cells remained undifferentiated or partially differentiated in this niche [98]. In an interesting study, a mixed population of NRPs and GRPs were transplanted into the injured spinal cord. The mixed population was provided by either direct isolation from fetal spinal cord or pre-differentiation of NSCs *in vitro*. This approach resulted in generation of a microenvironment that led to an excellent survival, migration out of the injury site and differentiation of the cells into both neural and glial phenotypes [99,100]. Functional improvements have been reported after transplantation of NSCs derived from embryonic spinal cord [85] and brain [101], adult brain [102] and spinal cord [103], and a mixed population of NRPs and GRPs isolated from fetal spinal cord [104].

### Hematopoietic stem cells and marrow stromal cells

As hematopoietic stem cells (HSCs) and marrow stromal cells (also known as mesenchymal stem cells) (MSCs) are more accessible than other cells mentioned in this review, they have attracted much attention as the potential cell sources in management of spinal cord injury. Bone marrow is a rich source of these cells; although, HSCs have also been obtained from umbilical cord blood [105] and fetal tissues [106].

Much of the evidence used to support the potential of HSCs and MSCs to differentiate into neural and glial cells comes from *in vivo *studies. Transplantation of unfractioned bone marrow has led to detection of bone marrow-derived cells that expressed neural markers in CNS, in both animal models [107-109] and humans [110,111]. In a recent clinical trial [112] bone marrow cells were delivered to patients with acute and chronic SCI intravenously or via vertebral artery. The study demonstrated the safety of the procedure. Partial improvement in the ASIA score and partial recovery of electrophysiological recordings of motor and somatosensory potentials have been observed in all subacute patients (n = 4) who received cells via vertebral artery and in one out of four subacute patients who received cells intravenously. Improvement was also found in one out of two chronic patients who received cells via vertebral artery. In another clinical trial unfractioned bone marrow cells were transplanted in conjunction with the administration of granulocyte macrophage-colony stimulating factor (GM-CSF) in six complete SCI patients and followed for 6–18 months. The procedure was safe and led to sensory improvements immediately. Also, AIS scores improved in 5 patients [113].

As unfractioned bone marrow is a mixture of different progenitor cells that might show different behavior in the same condition, more detailed studies have been performed on isolated fractions of HSCs and MSCs. Derivation of cells which have been phenotypically defined as neurons [106,114] and glial cells [105,106] has been reported after *in vitro *differentiation of HSCs. But, the point to be remembered is the fact that subsets of hematopoietic stem cells express neuronal and oligodendroglial marker genes [115,116] and this should be considered in interpretation of results of any differentiation study.

It was reported that transplanted hematopoietic stem cells transdifferentiate *in vivo *into neurons and glial cells without fusion [117]. But, dissimilar results were obtained from *in vivo *transdifferentiation studies. For example Koshizuka *et al *[118] have shown that HSCs only differentiate into glial cells not neurons. Lack of transdifferentiation into neurons, which is a matter of controversy [119-121], was also reported by Wagers *et al *[122] and Castro *et al *[123]. A recent electrophysiological study on neuron-like cells derived from HSCs failed to detect generation of action potentials in these cells [124]. But, locomotor improvement has been reported in the mice with contused spinal cord after transplantation of hematopoietic stem cells [118,125]. Also, it was shown that implantation of HSCs into developing spinal cord lesion of chicken embryos directs these cells to differentiate into neurons with no apparent fusion to the host cells [126]. These apparently disparate findings may be due to the issues such as the employed technique, the subpopulation of the HSCs used, and the experimental model. A phase I clinical trial in which CD34+ cells were delivered into the injured spinal cord via lumbar puncture technique demonstrated feasibility and safety of the procedure after 12 weeks of follow up [127].

The capacity of marrow stromal cells (MSCs) to differentiate *in vitro *into cells expressing neuronal markers have been shown in a number of studies [128,129], and the potential of these cells to generate voltage-sensitive ionic current was confirmed by electrophysiological recording [130]. *In vitro *differentiation into glial cells was also reported [131]. *In vivo *differentiation into neurons [132,133] and glial cells [134-136] has been reported in a number of studies. But a few studies have failed to demonstrate this transdifferentiation [125,137,138]. Fusion is another observation that needs to be considered. The question that bone marrow cells may adopt the phenotype of other cells by cell fusion was raised by *in vitro *observations [139,140] and tested in an *in vivo *model in which fusion of marrow stromal cells with Purkinje neurons was detected [141]. It has been shown that transplanted cells are capable not only to migrate in the injured tissue [135,142] but also to attract host cells to the site of transplantation [137]. Also, they form cell bridges within the traumatic cavity [134,137]. To address the best rout of delivery of these cells, chronic paraplegic rats received MSCs either locally or intravenously and it was concluded that transplantation of the cells to the spinal cord leads to superior functional recovery [143]. Locomotor improvements have been reported in most of the above studies even in those that did not detect transdifferentiation. This observation was attributed to secretion of cytokines and growth factors from MSCs [138,144], which might be subjected to batch-to-batch variation [138]. The point to be considered is that in most studies locomotor function was assessed by the Basso-Beattie-Breshnahan (BBB) test, which is a subjective test. More objective tests such as electrophysiological studies should be considered for achieving to more conclusive results. To the author's knowledge, no peer-reviewed clinical trial using MSCs for SCI patients has been published yet. But, a clinical trial involving transplantation of *in vitro *expanded MSCs to the spinal cord of the patients with amyotrophic lateral sclerosis revealed that the procedure is safe and feasible [145].

### Embryonic stem (ES) cells

Embryonic stem (ES) cells are pluripotent cells derived from inner cell mass of the blastocyst, an early embryonic stage. It has been known for many years that pluripotent embryonic stem cells can proliferate indefinitely *in vitro *and are able to differentiate into derivatives of all three germ layers [146].

Neural stem cells derived from ES cells can lead to behavioral improvement after transplantation to the site of injury in the spinal cord [147]. It has been shown that after prolonged *in vitro *expansion of ES cells-derived neural stem cells, they remain able to differentiate into neurons and astrocytes both *in vitro *and upon transplantation into brain [148]. Transplantation of motor neuron-committed ES cells to the injured spinal cord combined with pharmacological inhibition of myelin-mediated axon repulsion and provision of attractive cues within the peripheral nerves led to extension of transplanted axons out of the spinal cord. The axons reached the muscle, formed neuromuscular junctions and their functionality was confirmed by electrophysiological studies [149]. Transfection of ES cells with MASH1 gene is another strategy that caused ES cells to differentiated into motor neurons lacking Nogo receptor after transplantation into the transected spinal cord of mice and led to functional improvements confirmed by electrophysiological assessment [150]. Myelination was also addressed in a number of studies; for example, it was shown that neural cells derived *in vitro *from ES cells can myelinate the demyelinated rat spinal cord upon transplantation [151]. Oligodendrocyte-restricted progenitor cells were also derived from ES cells and were able to enhance remyelination and led to functional improvements after transplantation into a rat model of acute spinal cord injury [152].

## Scaffolds

As, lack of extracellular matrix at lesion site that directs and organizes the wound healing cells is one of the mechanisms that interferes with regenerative process after spinal cord injury, different studies have been conducted to investigate the potential of bioscaffold grafts to promote regeneration in the injured spinal cord, and to provide a bridge through which the regenerating axons can be properly guided from one end of the injury to the other end. Scaffolds were applied either alone or, to increase their healing effects, in combination with different growth factors or cellular components.

## Acellular scaffolds

### Collagen

As the major constituent of extracellular matrix, collagen supports neural cells attachment and growth [153]. NeuraGen™ Nerve Gide, a commercial peripheral nerve graft made of type I collagen, received FDA clearance for marketing in 2001. In spinal cord injuries, collagen has been used to fill the gap and the present evidence shows that it supports axonal regeneration. Collagen is a component of inhibitory glial scar and there is some evidence that it might inhibit nerve growth [154]. But, it has been suggested that collagen is not inhibitory to axonal regeneration per se and its effects depend on whether it contains inhibitory or trophic factors (see Klapka and Müller [155] for review). Application of cross-linked collagen and collagen filaments [156,157] have been studied in animal models of SCI. They increased regenerative activity in the spinal cord and improved the functional disability. It was observed that if the orientation of the grafted collagen fibers was parallel to the axis of the spinal cord, they promoted the growth of the regenerating axons into the graft from both proximal and distal ends. In this model, regenerating axons were also observed parallel to the axis of implant at the proximal host-implant interface. But, at the distal interface the running regenerating axons were entangled [156,157]. The results of implantation of a collagen tube in the injured spinal cords of rats were also promising showing that regenerating spinal axons regrow into the ventral root through this tube [158]. It has also been shown that impregnation of collagen with neurotrophin-3, increased the growth of corticospinal tract fibers into the implant and led to significant recovery of function of rats under investigation despite absence of regrowth of these fibers into the host tissue [159]. Surgical reconstruction of transected cat spinal cord using collagen plus omental transposition increased regenerative activity and led to functional recovery [160]. Functional recovery has also been observed by collagen implantation and omental transposition in a patient with SCI [160]. It has been shown that inclusion of collagen, supplemented with fibroblast growth factor-1 (FGF-1) or neurotrophin-3 (NT-3), within the hydrogel guidance channels improves axonal regeneration. FGF-1 increases axonal regeneration from reticular and vestibular brainstem motor neurons. But, NT-3 decreases the regeneration rate of brainstem motor neurons and only increases local axonal regeneration [154].

### Alginate

Alginate is an extracellular matrix derived from the brown seaweed from which a sponge has been developed by cross-linking of its fibers with covalent bonds [161]. In an *in vitro *study, it has been shown that when olfactory ensheathing cells, Schwann cells and bone marrow stromal cells are cultured on alginate hydrogel, they are transformed into atypical cells with spherical shape and their metabolic activities are inhibited; it has also been shown that alginate inhibits growth of dorsal root ganglia neurons [162].

But, when alginate sponge was implanted in the spinal cord of rats, it promoted axonal elongation, and the axons establish electrophysiologically functional projections and lead to functional improvements [163,164]. Also, interestingly, it was found that the axons that entered the sponge from the rostral and caudal stumps were able to leave the sponge from the opposite side and establish functional synapses with local neurons [165]. When compared with collagen, alginate reduced glial scar formation at the construct-tissue interface [161]. Also, the number of axons entered the alginate sponge were significantly higher than collagen [161]. In another experiment, alginate and fibronectin were used to coat poly-β-hydroxybutyrate (PHB) fibers obtained from bacterial cultures. When this construct was implanted to the rats with SCI, it increased the survival rate of rubrospinal tract axons. But, it did not lead to ingrowth of nerve fibers into the construct [166]. Recently, an alginate-based anisotropic capillary hydrogel (ACH) was implanted into the cervical spinal cord injury of rats and robustly increased the ingrowth of longitudinally directed regenerating axons into this implant [167].

### Poly(α-hydroxy acids)

Poly(α-hydroxy acids) are synthetic biodegradable polymers with excellent biocompatibility and the possibility of changing their specifications, and especially their mechanical properties and degradation rates, by alteration of the composition and distribution of their repeating units [168]. The advantages of synthetic scaffolds over the natural scaffolds are their lower batch-to-batch variation, more predictable and reproducible mechanical and physical properties and higher potential for control of materials impurities. It has been shown when the poly(_D, L_-lactic-co-glycolic acid) 50:50 (PLA_25_GA_50_) is applied to the completely transected spinal cord of rats, it demonstrates good mechanical properties and encourages axonal regeneration. The regenerated axons were observed penetrating the graft and the glial and inflammatory response near the lesion was similar to the controls [169]. For provision of a better 3-dimensional construct, macroporous scaffolds (foams) were made of poly(_D, L_-lactic acid) (PDLLA) containing poly(ethylene oxide)-block-poly(_D, L_-lactide) (PELA) copolymer (PDLLA-PELA foams). The foams were molded into small diameter rods and 14–20 rods were assembled using acidic fibroblast growth factor (aFGF)-containing fibrin glue and used to bridge the transected rat spinal cord. The construct was invaded by blood vessels and axons from proximal and distal spinal stumps, and axonal regrowth preferentially occurred along the main pore direction [170,171]. In another experiment, the same foam was made with the same diameter as rat spinal cord, treated with the neuroprotective brain-derived neurotrophic factor (BDNF), and embedded in fibrin glue containing aFGF. Apart from easier handling, this construct possessed a good flexibility and was able to support formation of blood vessels and migration of astrocytes, Schwann cells, and axons. BDNF led to the ingrowth of more regenerating axons to the implant, mainly at the rostral part. But the implants did not improve functional performance [172].

### Synthetic hydrogels

Synthetic hydrogels, such as poly [N-2-(hydroxypropyl) methacrylamide] (PHPMA) hydrogel (NeuroGel™) [173] and poly(2-hydroxyethyl methacrylate-co-methyl methacrylate) (PHEMA-MMA) [174], consist of crosslinked networks of hydrophilic co-polymers that swell in water and provide three-dimensional substrates for cell attachment and growth. Their ability to retain substantial amount of water with respect to the network density makes them suitable for transport of small molecules. These materials show low interfacial tension with biological fluids and can be formulated to have the same mechanical properties similar to the spinal cord [175-177]. They are nonbiodegradable materials. The advantage of these materials over the biodegradable materials is that they do not expose the tissues to the intermediary breakdown products, which may adversely affect the regeneration process [175].

After implantation of NeuroGel into the transected cat spinal cord, it was infiltrated by blood vessels, glial cells and regenerating descending supraspinal axons of the ventral funiculus and afferent fibers of the dorsal column, and most of regenerating axons were myelinated, mainly by Schwann cells. The regenerating axons were able to leave the implant both rostrally and caudally. The animals showed variable degrees of locomotor improvements [177]. Hydrogel decreased the gliotic scar formation at the interface between cord stump and the implant. Also, it considerably reduced the damage to the distal cord stump manifested by presence of more intact myelinated fibers and reduction of myelin degradation [178]. NeuroGel was also implanted in the post-traumatic lesion cavity in a rat model of chronic compression-produced injury of spinal cord. The hydrogel was invaded by blood vessels and glial cells. Also, ingrowth of regenerating axons was observed from the rostral stump into the NeuroGel. The axons were associated with well-organized myelin sheets and Schwann cells. Functional recovery was also observed [179]. In another interesting study, the cell-adhesive sequence Arg-Gly-Asp (RGD) of the central-binding domain of the extracellular matrix (ECM) glycoprotein fibronectin was incorporated into the NeuroGel (PHPMA-RGD hydrogel). This core tripeptide sequence plays a central role in the adhesion-mediated cell migration required for tissue construction during development and repair. The PHPMA-RGD hydrogel was implanted in the transected cord of rats and led to angiogenesis and axonal growth. It was shown that axons enter the construct from the rostral cord and leave it into the caudal stump. The axons were myelinated by Schwann cells, and supraspinal axons and synaptic connections were observed in the reconstructed cord segment. The rats showed some degrees of functional improvements [180].

PHEMA has a lower volume fraction compared with NeuroGel. When both NeuroGel and PHEMA were implanted into the rat cortex, NeuroGel was invaded by various connective tissue elements, but PHEMA hindered ingrowth of connective tissue and only allowed astrocyte invasion [181]. Unfilled PHEMA-MMA channels were used to bridge the transected spinal cord of rats using fibrin glue. A tissue bridge formed inside the channel between two stumps and brainstem motor neurons regenerated through this bridge to the distal stump. Also, the channel limited the ingrowth of scar tissue. But, the channels did not improve the functional recovery [174]. In another experiment, PHEMA soaked in brain-derived neurotrophic factor (BDNF) solution was implanted in hemisected rat spinal cords. BDNF did not have any effect on the scarring and angiogenesis but, it promoted axonal regeneration [175]. Axonal regeneration into the implant is also improved when PHEMA-MMA channels are filled with the matrices such as collagen, fibrin and Matrigel [154].

### Polyethylene glycol

Polyethylene glycol (PEG) is a water-soluble surfactant polymer. Brief application of aqueous solution of this polymer to the site of injury in the spinal cord seals and repairs cell membrane breaches, reverses the permeabilization of the membrane produced by injury, inhibits production of free radicals [182-184], and decreases oxidative stress [185,186]. PEG was able to re-establish the anatomical continuity and lead to functional recovery of severed guinea pig spinal cord [187]. It has been shown that brief application of PEG to the injured spinal cord of guinea pigs reduces cystic cavitation and the extent of the injury [188], and improves behavioral function [189,190]. But, prolonged application can induce conduction block [191].

### Fibrin

Fibrin is derived from blood and is the major component of clots. Fibrin functions as bridging molecule for many types of cell-cell interactions. At the site of injury, many cells directly bind to the fibrin via their surface receptors. This helps localization of these cells to the site of injury and carrying out their specialized function [192]. In the treatment of SCI, the fibrin is usually enriched with acidic fibroblast growth factor (aFGF) and is used in conjunction with other modalities. Its application in combination with poly(α-hydroxy acids) and synthetic hydrogels has been described in the above paragraphs. When the site of cord injury was filled with a fibrin gel, which was engineered to release neurotrophin-3 after degradation by the invading cells, vigorous cellular infiltration of the fibrin and diminished formation of the glial scar was observed [193]. In addition to the above applications, fibrin glue is regularly used for stabilization of cellular bridges to the implantation site (see below).

### Matrigel

Matrigel is an extracellular matrix extracted from the Engelbreth Holm Swarm (EHS) sarcoma and contains laminin, fibronectin, and proteoglycans, with laminin predominating [194]. In an *in vitro *study, it has been shown that Matrigel stimulates cell proliferation and preserves the typical morphological features of olfactory ensheathing cells, Schwann cells and bone marrow stromal cells in culture; and it also supports growth of dorsal root ganglia neurons [162]. Implantation of Matrigel alone does not increase regenerative activities in the spinal cord [195]. But, Matrigel combined with vascular endothelial growth factor (VEGF) or a replication-defective adenovirus coding for VEGF decreases retrograde degeneration of corticospinal tract axons and increases axonal regenerative activities in rats. Regenerating axons growing from the rostral part of the lesion cross the implant and can be found in the distal cord [196]. Also, inclusion of Matrigel within hydrogel guidance channels increases the number of regenerating axons penetrating the construct. But, this inhibits regeneration of brainstem motor neurons [154]. Also, it has been shown that implantation of PAN/PVC guidance channels (see below) containing Matrigel enriched with glial cell line-derived neurotrophic factor (GDNF) enhances growth of regenerating axons into the implant [197]. Matrigel has been used as regular scaffold for construction of bridges made of Schwann cells and also for delivery of human adult olfactory neuroepithelial-derived progenitors (see below).

### Fibronectin

Fibronectin (Fn) is a glycoprotein found in many extracellular matrices and in plasma. It is involved in cell attachment and migration due to its interaction with cell surface receptors [198]. Fibrous aggregates of plasma fibronectin have been used to make fibronectin mats. These mats contain pores oriented in a single direction [199]. The rate of resorption of these mats can be modified by incorporation of copper and zinc ions [200]. When Fn mats were implanted in hemisected rats spinal cords, they well integrated with the spinal cord and showed little cavitation either within or adjacent to the implant. Orientated growth of GABAergic, cholinergic, glutamatergic, noradrenergic axons and calcitonin gene-related peptide (CGRP)-positive neurons occurred into the mat and axons were myelinated by Schwann cells. Incubation of mats with BDNF and NT-3 increased neurofilament-positive and glutaminergic fibers. Incorporation of nerve growth factor into the mats increased the number of CGRP-positive neurons. But, there was little axonal outgrowth from the mats into the host spinal cord [199]. After implantation, Fn mats are vascularized and infiltrated by macrophages, axons and Schwann cells that myelinate the axons, oligodendrocytes and their precursors and astrocytes. Laminin deposition is also observed in the mats [201]. This failure of outgrowth of axons from the mat to the surrounding tissue was attributed to the astrocytosis and glial scar formation around the implant. The attempts to decrease this astrocytosis by incubation of mats with antibodies to transforming growth factor β (TGFβ) not only did not solve the problem, but also exacerbated the extent of secondary damage [202].

In an *in vitro *study, it has been shown that combination of fibronectin with alginate hydrogel supports olfactory ensheathing cells proliferation. But, the proliferation rate was significantly lower than what was observed on Matrigel [162]. Incorporation of the central binding domain of fibronectin i.e. Arg-Gly-Asp (RGD) to the NeuroGel (PHPMA-RGD hydrogel) has been performed in an interesting study to enhance its cell adhesion and guidance capacity. Implantation of this construct into the spinal cord of rats led to angiogenesis and axonal growth into the implant (see above) [180]. Fibronectin has also been used to make fibronectin cables with parallel fibril alignment. It has been shown that these cables support Schwann cells growth *in vitro *and these cells align with the axis of the fibrils [198].

### Agarose

Agarose is a polysaccharide derived from seaweed. Recently, a freeze-dried agarose scaffold with uniaxial linear pores extending through its full length was manufactured and its biocompatibility and ability to function as a depot for growth factors was confirmed by *in vitro *studies [203]. These scaffolds retain their microstructure without the use of chemical cross-linkers. Also, they can retain their guidance capabilities within the spinal cord for at least 1 month. Implantation of BDNF-incorporated scaffolds in a rat model of spinal cord injury, led to organized and linear axonal growth into the agarose. The implant was also penetrated with Schwann cells, blood vessels and macrophages. Agarose did not evoke fibrous tissue encapsulation in host tissue [204].

Another recent approach is to use *in situ *gelling agarose hydrogel. An irregular, dorsal over-hemisection spinal cord defect in adult rats was filled with agarose solution embedded with BDNF-loaded microtubules and was cooled until gelation. This allowed the gel to conformally fill the defect by adopting its shape and minimized the gap between tissue and scaffold. The implant was penetrated by axons only in the presence of BDNF. But, no outgrowth of axons from the implant to the host distal cord was observed. The other observed effect was reduction of the intensity of reactive astrocytosis and deposition of chondroitin sulfate proteoglycans (CSPGs) by BDNF [205].

## Cell-scaffold constructs

### Matrigel constructs

Matrigel has been used as a scaffold for *in vivo *delivery of Schwann cells in several experiments. Purified Schwann cells were mixed with Matrigel and inserted in semipermeable non-degradable 60/40 polyacrylonitrile/polyvinylchloride (PAN/PVC) copolymer guidance channels. This construct was used to bridge a transected rat spinal cord. Histological studies demonstrated penetration of the implanted bridge by myelinated axons, blood vessels, macrophages and fibroblasts. When the models underwent electrophysiological studies, stimulus-evoked cord potentials were clearly identified in a few models, showing functionality of regenerating axons [70]. When this model was combined by infusion of BDNF or NT-3 to the distal cord stump, axonal growth from the implant into the distal host spinal cord stump was effectively promoted for several cord segments. In the absence of BDNF or NT-3 only a few axons were able to enter the distal stump [78]. In another experiment, instead of infusion of BDNF distal to the implant, the BDNF was added to the SC/Matrigel cable inside the PAN/PVC guidance channels. This approach led to increased growth of regenerating axons into the construct as well. Also, GDNF decreased the extent of reactive gliosis and cystic cavitation at the graft-host interface [197]. Recently, a combination of SC/Matrigel cable inside PAN/PVC channels with implantation of olfactory ensheathing cells (OECs) in the distal and proximal cord stumps and infusion of chondroitinase ABC to the SC bridge/host spinal cord interface was studied in a rat model of spinal cord transection [79]. OECs were implanted to enable regenerating axons to exit the SC/Matrigel bridge, and chondroitinase ABC was used to reduce the axonal regeneration inhibitory effect of chondroitin sulfate proteoglycan (CSPG) in the glial scar. This combined implantation therapy significantly increased the number of myelinated axons and serotonergic fibers in the bridge, and the latter grow in the distal cord stump. Also, significant functional improvement was observed. In another experiment carried out by implantation of SC/Matrigel cables contained in biodegradable scaffolds made of poly(alpha-hydroxy acids) (PHAs) such as poly(_D, L_-lactic acid) (PLA_50_) or high molecular weight poly(_L_-lactic acid) mixed with 10% poly(_L_-lactic acid) oligomers (PLA_100/10_), the intervention led to axonal ingrowth into the implant but, it was not as effective as the PAN/PVC experiment [206]. In another experiment, Matrigel was used for seeding of Schwann cells derived from human bone marrow stromal cells in an ultra-filtration membrane (Millipore) tube. This construct promoted axonal regeneration into the bridge and resulted in recovery of hind limb function in rats [207].

Recently, the potential of delivering human adult olfactory neuroepithelial-derived progenitors with Matrigel was studied in a rat model of hemisected spinal cord injury. This approach has led to regeneration of rubrospinal neurons through the transplant within the white matter for several segments caudal to the graft so that a few rubrospinal axons terminated in gray matter close to motor neurons. Improvements in functional recovery were also observed in this experiment [195].

### Collagen construct

The ease of manipulation of collagen into various shapes allows precise application of the cells to the injured site. Cortical neonatal rat astrocytes were embedded in collagen type I gel and transplanted to the hemisected rat spinal cords. Collagen prevented migration of astrocytes into the host tissue, which was believed to be an advantage, as their presence could attract more regenerating axons into the implant. This approach has resulted in significant increase of number of ingrowing neurofilament-positive fibers (including corticospinal axons) into the implant. But, the fibers did not reenter the host tissue. Modest temporary improvements of locomotor recovery were observed in this study which was hypothetically attributed to the factors secreted from transplanted astrocytes [208].

### Alginate constructs

Recently, it has been shown that adult neural progenitor cells harvested from rats cervical spine can be mounted on an alginate-based anisotropic capillary hydrogel (ACH) and this construct supports axonal regeneration *in vitro *[167].

In another experiment, neurospheres prepared from fetal rat hippocampus were injected into the alginate sponge, and implanted in the injured spinal cord of rats. Alginate increased the survival of neurospheres after transplantation and supported their migration, differentiation and integration to the host spinal cord [209]. Microencapsulation of fibroblasts producing brain-derived neurotrophic factor (BDNF) in alginate-poly-_L_-ornithine is another method for application of alginate in treatment of SCI. Microcapsules protect fibroblasts from the host immune response and eliminate the need for immunosuppressive therapy. These constructs were injected to the spinal cord in a rat model of SCI and promoted growth of regenerating axons into the cellular matrix that developed between the capsules. They also led to improvement of the function of the affected limbs [210,211]. In another study, neonatal Schwann cells were seeded on alginate and fibronectin-coated poly-β-hydroxybutyrate (PHB) fibers and supported ingrowth of regenerating axons, which extended along the entire length of the graft [166].

### Fibrin constructs

Fibrin has been used to enhance the effects of cell-scaffold constructs. In most instances, fibrin is used with acidic fibroblast growth factor (aFGF). It has been shown that basic fibroblast growth factor (bFGF) is not efficient in this setting [212]. Fibrin containing aFGF has been applied to both ends of Schwann cells/Matrigel cables in PAN/PVC guidance channels. They increased sprouting of corticospinal tracts in rats; and the axons that entered the graft left the implant and entered the host spinal cord from the opposite end [213]. Preparation of a mixture of cell suspension and fibrinogen for direct transplantation to the injured spinal cord is another approach for application of fibrin in clotted form. But, such a preparation made of olfactory ensheathing cells (OECs) did not prove to be effective in a rat model of SCI [45]. Fibrin clots have been used for delivery of Schwann cells as well. SC/fibrin clot has been inserted in PAN/PVC guidance channels and were used to bridge a transected rat spinal cords. This was combined by transduction of caudal spinal cord stump cells with adeno-associated viral (AAV) vectors encoding for brain-derived neurotrophic factor (BDNF) or neurotrophin-3 (AAV-NT-3). Histological sections have shown the ingrowth of axons from the rostral stump into the bridge, but the axons did not leave the bridge. On the other hand, the transduced neurons in the caudal stump extended their processes into the implant. This combined treatment led to significant improvement of hind limb function in treated animals [214].

### Poly(α-hydroxy acids)-construct

A two-component scaffold was made of a blend of 50:50 poly(lactic-co-glycolic acid) (PLGA) (75%) and a block copolymer of poly(lactic-co-glycolic acid)-polylysine (25%). The scaffold's inner portion emulated the gray matter via a porous polymer layer and its outer portion emulated the white matter with long, axially oriented pores for axonal guidance and radial porosity to allow fluid transport while inhibiting ingrowth of scar tissue. The inner layer was seeded with a clonal multipotent neural precursor cell line originally derived from the external germinal layer of neonatal mouse cerebellum. Implantation of this construct into the hemisection adult rat model of spinal cord injury led to a long-term functional improvement accompanied by reduction of epidural and glial scar formation and growing of regenerating corticospinal tract fibers through the construct, from the injury epicenter to the caudal cord [215].

## Conclusion

The complicated pathophysiology of spinal cord injury and its consequent disability had made the pace of therapeutic interventions in this field very slow for many years. But, in the last decade, the rapid progress that has been made in the field of tissue engineering as the result of advances made in areas of cell biology and biomaterials, opened up the way for new therapeutic strategies. These new strategies have shown promising results and the scientists are hoped to cure the patients with spinal cord injury before long.

## Competing interests

The author(s) declare that they have no competing interests.

## Authors' contributions

Single author
